# Effect of Global Cardiac Ischemia on Human Ventricular Fibrillation: Insights from a Multi-scale Mechanistic Model of the Human Heart

**DOI:** 10.1371/journal.pcbi.1003891

**Published:** 2014-11-06

**Authors:** Ivan V. Kazbanov, Richard H. Clayton, Martyn P. Nash, Chris P. Bradley, David J. Paterson, Martin P. Hayward, Peter Taggart, Alexander V. Panfilov

**Affiliations:** 1 Department of Physics and Astronomy, Ghent University, Ghent, Belgium; 2 INSIGNEO Institute for In-Silico Medicine, University of Sheffield, Sheffield, United Kingdom; 3 Department of Computer Science, University of Sheffield, Sheffield, United Kingdom; 4 Auckland Bioengineering Institute, University of Auckland, Auckland, New Zealand; 5 Department of Engineering Science, University of Auckland, Auckland, New Zealand; 6 Department of Physiology, Anatomy and Genetics, University of Oxford, Oxford, United Kingdom; 7 Departments of Cardiology and Cardiothoracic Surgery, University College Hospital, London, United Kingdom; 8 Moscow Institute of Physics and Technology (State University), Dolgoprudny, Moscow Region, Russia; Université Bordeaux Segalen, France

## Abstract

Acute regional ischemia in the heart can lead to cardiac arrhythmias such as ventricular fibrillation (VF), which in turn compromise cardiac output and result in secondary global cardiac ischemia. The secondary ischemia may influence the underlying arrhythmia mechanism. A recent clinical study documents the effect of global cardiac ischaemia on the mechanisms of VF. During 150 seconds of global ischemia the dominant frequency of activation decreased, while after reperfusion it increased rapidly. At the same time the complexity of epicardial excitation, measured as the number of epicardical phase singularity points, remained approximately constant during ischemia. Here we perform numerical studies based on these clinical data and propose explanations for the observed dynamics of the period and complexity of activation patterns. In particular, we study the effects on ischemia in pseudo-1D and 2D cardiac tissue models as well as in an anatomically accurate model of human heart ventricles. We demonstrate that the fall of dominant frequency in VF during secondary ischemia can be explained by an increase in extracellular potassium, while the increase during reperfusion is consistent with washout of potassium and continued activation of the ATP-dependent potassium channels. We also suggest that memory effects are responsible for the observed complexity dynamics. In addition, we present unpublished clinical results of individual patient recordings and propose a way of estimating extracellular potassium and activation of ATP-dependent potassium channels from these measurements.

## Author Summary

Cardiac arrhythmias are an important cause of death in the industrialized world. The most dangerous type of cardiac arrhythmias is ventricular fibrillation. If left untreated, it leads to death within just few minutes. In most of the cases ventricular fibrillation occurs as a result of cardiac ischemia, which is a shortage of blood supply to the heart muscle. Futhermore ventricular fibrillation leads to decreased cardiac output, which in turn results in secondary ischemia. A recent clinical study investigated the effect of secondary ischemia on the organization of ventricular fibrillation in the human heart. However, in the clinical study it was not possible to obtain the whole picture of activation of the heart and to separate the relative roles of different processes induced by ischemia in the alterations to the cardiac electrical activity. In this study we use computer modeling to address these problems and to complement the results of the clinical study. Our results allow us to explain the change of electrical activation pattern in the heart during the first minutes of ischemia and to estimate the relative rates of those ischemia-induced physiological processes. We also present previously unpublished data on individual patient recordings from the clinical study.

## Introduction

The heart is an electromechanical pump, where contraction is triggered and synchronized by electrical activation originating from the sinoatrial node. Abnormal initiation or conduction of the electrical impulses could result in a cardiac arrhythmia. Cardiac arrhythmias are an important cause of sudden and premature death in the industrialized world. In many cases the lethal event is ventricular fibrillation (VF). During VF, rapid and self-sustaining electrical activity in the ventricles acts to suppress the natural pacemaker, resulting in uncoordinated, weak and rapid contractions, which lead to death within several minutes [1].

VF often occurs as a result of acute regional cardiac ischemia, which is a condition when blood flow to part of the heart is substantially decreased, for example by reduced flow through a coronary artery [2]. In addition, global ischemia unavoidably accompanies VF, because the abrupt fall in cardiac output resulting from VF also results in compromised myocardial perfusion. Thus an episode of spontaneous VF will result in a progressively ischemic heart. The effect of this secondary ischemia on electrical activity during VF is important clinically, because defibrillation typically occurs several minutes after the onset of VF, and thus the mechanism is likely to have been modified by ischemia [3], [4].

It is known that ischemia profoundly affects the electrophysiological properties of cardiac cells and tissue [5]. During VF, the rapidly changing patterns of electrical activity in the ventricles are sustained by re-entry, in which waves of electrical activation continually propagate into regions of recovered tissue [6]. Re-entry is seen as a spiral wave on the surface of the heart, and a scroll shaped activation wave in 3D cardiac tissue [7]. The question of how ischemia influences the behaviour of re-entrant activity during VF is important, and has been addressed by clinical, experimental and modeling studies.

Two important characteristics of VF are the frequency and spatiotemporal complexity of activation patterns, and these have been studied in animal heart experiments. Experiments on canine hearts [6] have shown that both activation rate and pattern complexity (measured as the number of wave fronts) increase slightly during the first minute of VF, followed by a decrease over periods of up to 10 minutes, associated with progressive global cardiac ischemia. Studies in porcine hearts, [8], demonstrated a monotonic decrease of the activation rate of VF during the first 5 minutes of ischemia, with a decrease in complexity for the first two minutes followed by a rapid increase.

In spite of the significance of these animal studies, the most valuable question for clinical practice is how global ischemia modulates the mechanism of VF in the human heart. Both clinical and modelling studies have established that the organization of VF in the human heart is quantitatively different to that in canine and porcine hearts [9], [10], with VF in the human heart shown to be characterized by a much lower VF complexity compared with animal hearts of similar size. For this reason, experimental studies on human hearts are extremely important for understanding underlying mechanisms. Recently this gap has partially been filled by studies in isolated myopathic hearts [11], [12], and studies in the in-situ human heart [13].

In the in-situ study [13], electrical activity was mapped on the heart surface during VF in ten patients undergoing routine cardiac surgery with cross-clamp fibrillation. Following the commencement of cardiopulmonary bypass to support the systemic circulation, the following protocol was used: (1) VF was induced by burst pacing, (2) after 30 s, global cardiac ischemia was initiated by applying an aortic cross-clamp, (3) after 2.5 min. ischemia, cardiac perfusion was restored by release of the cross-clamp, (4) recording continued for 30 s during reperfusion. During all 3.5 minutes of VF, electrical activity was recorded using 256 unipolar electrodes sewn into an elasticated sock placed over the entire ventricular epicardium by the surgeon. Consistent with studies in animal hearts, the activation rate of fibrillation gradually decreased during global ischemia and increased abruptly after reperfusion. In contrast with animal hearts, the complexity of fibrillation patterns (measured as the number of phase singularities) continued to increase gradually during ischemia, although the data showed large variation.

The above mentioned study [13] had several important limitations, in common with other studies in human and animal hearts. First, electrical activity was recorded only on the epicardial surface, whereas electrical activation patterns in the ventricles are 3-dimensional. Second, ischemia is a complex process which involves several separate mechanisms including hyperkalemia, hypoxia and acidosis, and each of these components has its own time course in ischemia [5]. However, it is not generally possible to measure the relative contribution of these individual components to the excitation patterns *in vivo*.

Because it is difficult to measure all of the quantities of interest, even in animal hearts and tissue, detailed mechanistic and multi-scale models of cardiac electrophysiology at cell, tissue, and whole organ scales are becoming important research tools for the study of arrhythmia mechanisms. It was shown previously that the combination of modeling with experimental and clinical studies can provide valuable insights into arrhythmia mechanisms [9], [10], [14].

In this paper, we describe a detailed and comprehensive modeling study, which seeks to establish mechanisms that are consistent with the changes in activation patterns observed during VF in the human heart with global ischemia [13]. In addition, we present previously unpublished clinical results from [13], namely the changes in dominant frequency (DF) for individual patients. The model we use is a representation of human ventricular tissue, with cellular electrophysiology described by the TNNP06 model [15]. This model was adapted to enable us to investigate the effects on VF of three components of ischemia [5] both separately and in combination. Briefly the effects of these three components on the eletrophysiological processes can be characterized as follows. Hyperkalemia is elevation of the extracellular potassium concentration, which leads to a shift of the resting potential of cardiac cells. Acidosis is an increase in intracellular pH, which results in smaller fast sodium and L-type calcium currents during depolarization. Hypoxia is a reduced oxygen supply, which impairs cellular metabolism and changes the ratio of ADP to ATP concentration inside the cell. In turn, this change results in the opening of specific ATP-dependent potassium channels. To describe them in our simulations, we developed a new model of the human 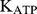 channel based on experimental data from human cardiac cells [16].

We first determined the effect of each of the three components of ischemia on action potential duration (APD) and conduction velocity (CV) restitution in 1D models, along with the effects on re-entry in 2D models. Then we performed simulations of VF using a 3D anatomical model of the human ventricles [10], where the dependency of the activation rate of VF on each component of ischemia was determined.

Next, we used these dependencies to interpret the clinical recordings for individual patients. We showed that the predominant factor responsible for the change in activation rate during ischemia is hyperkalemia, and estimated the magnitude of this effect in each individual patient recording. Then we studied how the three ischemic components affect the complexity of VF expressed by the number of scroll wave filaments. Although there was a big variation, the results we obtained were qualitatively similar to the clinical recordings and we concluded that the main factors important for the observed dynamics were memory effects. We also proposed a simple algorithm to estimate the dynamics of hyperkalemia and the activation of the 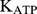 current using individual patient recordings. Overall, we demonstrated that taking into account the electrophysiological changes caused by hyperkalemia and hypoxia, the results of [13] can be fully explained, indicating that acidosis has only a minor effect on VF activation during the first few minutes of ischemia.

## Methods

### Electrophysiological model

Cardiac cellular electrophysiology was modeled using the TP06 model of human ventricular cardiomyocyte [15], [17] coupled into a monodomain model for cardiac tissue. In this model the main equation determining the propagation of transmembrane voltage is given by:
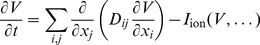
(1)where 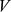 is transmembrane voltage, 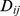 a diffusion tensor and 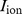 is the sum of ionic currents:
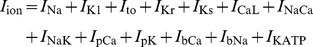
(2)where all currents except 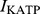 were described by the equations from [18], [15], and 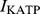 was a new current that we introduced to take the effects of hypoxia into account. For most parameters we used the values listed in Table 1 and Table 2 of [15] corresponding to epicardial cells. For the five parameters listed in Table 2 of [15] we used the values corresponding to “Slope 1.8” for 3D simulations, and “Slope 1.1” in 2D simulations for finding the restitution curves. We used parameters corresponding to all three different slopes: 1.1, 1.4 and 1.8, for analyzing the stability of re-entry in 2D.

**Table 1:** Summary of changes applied to the TNNP06 model.

Ischemia component	Modeled by	Within range
Hyperkalemia	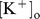	5–12 mM
Acidosis	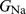 , 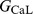	20–100% of base values
Hypoxia	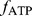	0–0.05%

**Table 2:** Estimated values of extracellular potassium concentration and number of activated 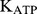 channels for the patients studied in [13].

	Regular fit						Alternative fit	
	0%		25%		50%		0%	
Patient	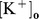	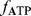	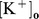	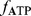	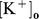	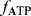	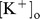	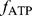
H055	7.2	0.024	7.3	0.032	7.5	0.049	7.2	0.076
H057	7.2	0.020	7.3	0.027	7.5	0.041	7.2	0.055
H058	7.6	0.029	7.7	0.039	7.9	0.058	7.9	0.141
H059	6.9	0.049	7.1	0.065	7.4	0.098	7.0	0.102
H060	7.0	0.031	7.1	0.041	7.3	0.062	7.0	0.053
H062	6.7	0.012	6.8	0.016	6.9	0.025	6.8	0.098
H063	7.4	0.010	7.4	0.013	7.5	0.020	7.3	0.076
H064	6.7	0.019	6.8	0.025	7.0	0.038	6.7	0.047
H065	8.0	0.072	8.2	0.096	8.8	0.144	8.4	0.157
H066	7.4	0.046	7.6	0.061	7.8	0.092	7.5	0.096

The estimate for the ‘regular fit’ is based on graphs from Figure 5 scaled to the value of the period at point A. For the ‘alternative fit’ we scaled our results to the value of the period given at the beginning of the clinical recordings. The percentages correspond to the ratios of 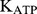 channels recovered during reperfusion. Further explanations are in the text.

Ischemia was introduced to the model by changing the parameters corresponding to each of the three ischemia components described above. To model hyperkalemia we varied 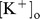 in the range 5–10 mM. Acidosis was taken into account by decreasing the maximum conductivity of sodium and L-type calcium currents within the limits of 20–100%. The effect of hypoxia was represented by a novel description of the ATP dependent potassium current in the human heart.

Table 1 summarizes the changes to the TP06 model due to ischemia.

### ATP-dependent potassium current

**Figure 1:**
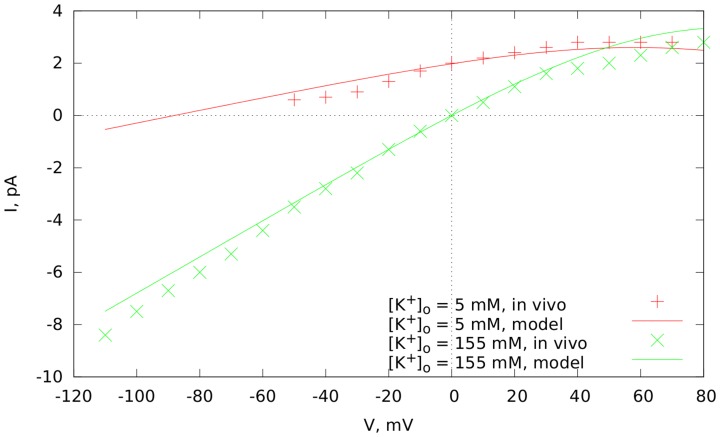
Current-voltage dependency for the 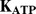 current in our model. The experimental data is from [16].

We developed our model of the 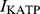 current based on the results of *in vitro* experiments [16]. Figure 1 shows measured current-voltage dependencies of 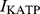 for two different extracellular potassium concentrations. We fitted these data using functions with a power dependency on 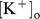 concentration and exponential functions of voltage, similar to functions used in [19], [20] for 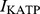. Our expression for human 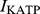 was:
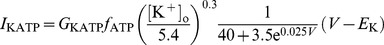
(3)where 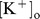 is in mM, 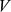 is transmembrane voltage measured in mV, and 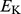 is the Nernst potential for potassium also in mV. Our function reproduces well the current-voltage dependency in a range between 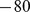 and 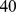 mV. We also see some small deviations for lower and higher values of voltage, which, however, should not be essential as they are outside the important physiological range.

The fraction of open 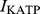 channels (or the probability for a channel to be open) is expressed by the factor 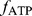. It depends on the energy capabilities of the cell. For our model we left this dependency unspecified, changing the value 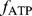 directly to induce hypoxia. The scaling coefficient 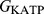 was fitted following the approach of [20], where the value of APD decreases twofold if 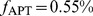 of channels are open, which led to 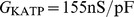.

### Numerical methods

For 1D and 2D simulations we solved equation (1) for homogeneous and isotropic tissue with the diffusion tensor 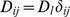 where 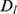 was taken to be 1.54 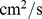 and 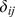 is the Kronecker delta. For these parameter values, the velocity of propagating plane waves at a stimulation frequency of 1 Hz was 72 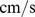.

For 3D whole heart simulations we used an anatomically based model of human ventricles presented in [21]. This model takes anisotropy into account by reconstructing the fiber direction field described in [22] and assuming that the diffusion coefficient across (transverse) the fibers 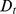 is 4 times less than the diffusion coefficient along fibers 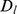, which was set to 1.54 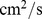. For 3D simulations the components of the diffusion tensor were given by:
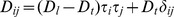
where 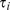 are the coordinates of a normalized vector oriented along the fibers.

For all types of the media we used ‘no flux’ boundary conditions:
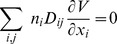
where 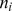 are the coordinates of a vector that is normal to the boundary.

To solve the differential equations we used a finite difference approach. For both 2D and 3D simulations we introduced a rectangular mesh of about one million points. To approximate the diffusion term we used a stencil of 5 grid points for 2D and 17 points for 3D. We used an explicit first order Euler method to solve the discretized system, which for 2D tissue was:
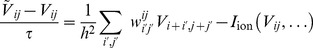
(4)where the time step 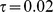 ms, 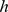 was the space step, and 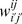 weights corresponding to the diffusion tensor at location 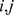. The space step was 0.25 mm for 2D simulations, and 0.5 mm for 3D simulations [23]. The gating variables in the TP06 model were integrated using the Rush and Larsen approach [24]. To cache the results returned by functions that were only voltage dependent we used pre-computed look-up tables.

The model was implemented using the C and C++ programming languages with OpenMP extensions for parallelization. We mainly used the Intel ICC compiler toolkit. The code for 2D was run on an Intel Core i7-3930K (3.20 GHz) machine, and the 3D code for human ventricles was run on dual-processor Intel Xeon E5-2650 (2.0 GHz) machines.

## Results

### APD and CV restitution

In a model of a thin strip of human cardiac tissue, we studied how the different components of ischemia influence APD and CV restitution, a dynamic property of cardiac tissue important for the onset of re-entry and for the stability of re-entrant waves [25], [26]. We followed the same approach as in [14], but with a different formulation of 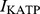.

**Figure 2:**
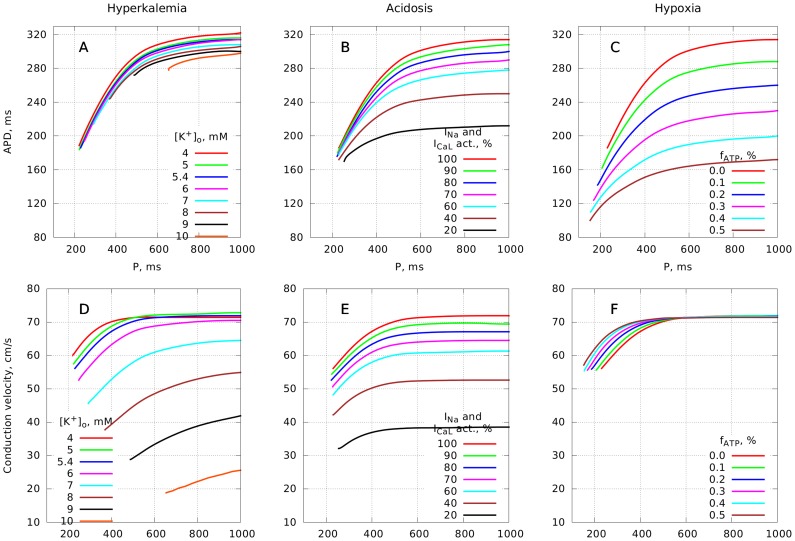
A, B, C: APD restitution curves for hyperkalemia, acidosis and hypoxia respectively. D, E, F: CV dispersion relations for hyperkalemia, acidosis and hypoxia respectively. 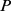 denotes the sum of diastolic interval (DI) and APD at the point of measurement for the pulse generated by the S2 stimulus.

Restitution and dispersion curves were obtained using an S1S2 protocol, in a 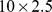 cm sheet of 2D simulated tissue. Superthreshold stimuli were delivered along one short edge of the sheet, and measurements of APD were made at a distance of 2.5 cm from the stimulated edge. The basic cycle length (BCL) for S1 stimuli was 1000 ms. The ten S1 stimuli were followed by a single S2 stimulus. The duration between the last S1 and S2 was decremented from 1000 ms until there was no response to the S2 stimulus at the point of measurement. The restitution curves are shown in Figure 2.

In Figure 2A the effect of hyperkalemia on APD restitution is shown, indicating that APD decreases with increasing 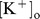. This effect is due to 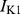 and 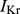 currents, which depend directly on 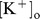 and become larger once 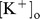 is increased, accelerating the repolarization process. The effect of hyperkalemia on conduction velocity is shown in Figure 2D. We see that the conduction velocity decreases with increasing 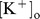. This is because an increase in 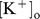 shifts the resting potential to more positive values suppressing the sodium current and, consequently, reducing the excitability of the cell.

Figures 2A and 2D also show how the minimal APD—
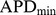
—value (the left most point of the restitution curve) depends on 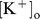. 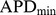 increases substantially with hyperkalemia. This fact can be important as 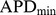 is considered as one of the main factors determining the complexity of pattern of excitation during fibrillation [21], [27].

Figures 2B and 2E illustrate the effect of acidosis (modeled as reduction of 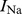 and 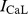) on APD and CV restitution. Both APD and CV decrease with acidosis, because acidosis diminishes the depolarizing currents available to the cell. However, the value of the 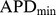 showed almost no dependency on acidosis, unless the conductance of the 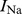 and 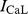 channels was reduced to 20% of their default values.

Figures 2C and 2F show the effect of hypoxia, modeled as activation of 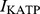. Both APD and 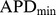 become substantially shorter since hypoxia activates a strong depolarizing current. The value of CV showed almost no dependency on hypoxia because hypoxia does not affect 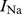.

Figure 2 shows that each component of ischemia acted to reduce the slope of restitution. Steep restitution is considered as one of the main mechanisms contributing to the breakup of re-entry in VF [25], [26]. According to the restitution hypothesis, flattening restitution prevents possible spiral wave breaks from happening, and therefore prevents formation of new arrhythmia sources.

### Effect of ischemia on arrhythmia sources in 2D

We studied how the different components of ischemia influence the dynamics and stability of re-entrant waves in 2D—spiral waves. In these simulations we varied the slope of the restitution curve for non ischemic conditions by changing five parameters of our model, as described in the Methods. We used three different sets of parameters, which correspond to a slope of 1.1, 1.4 and 1.8 under normal conditions. For initiation of a spiral we used an S1S2 protocol: at first, the S1 stimulus was delivered along one side of the medium, then the S2 stimulus was applied after the wave had passed half of the medium.

**Figure 3:**
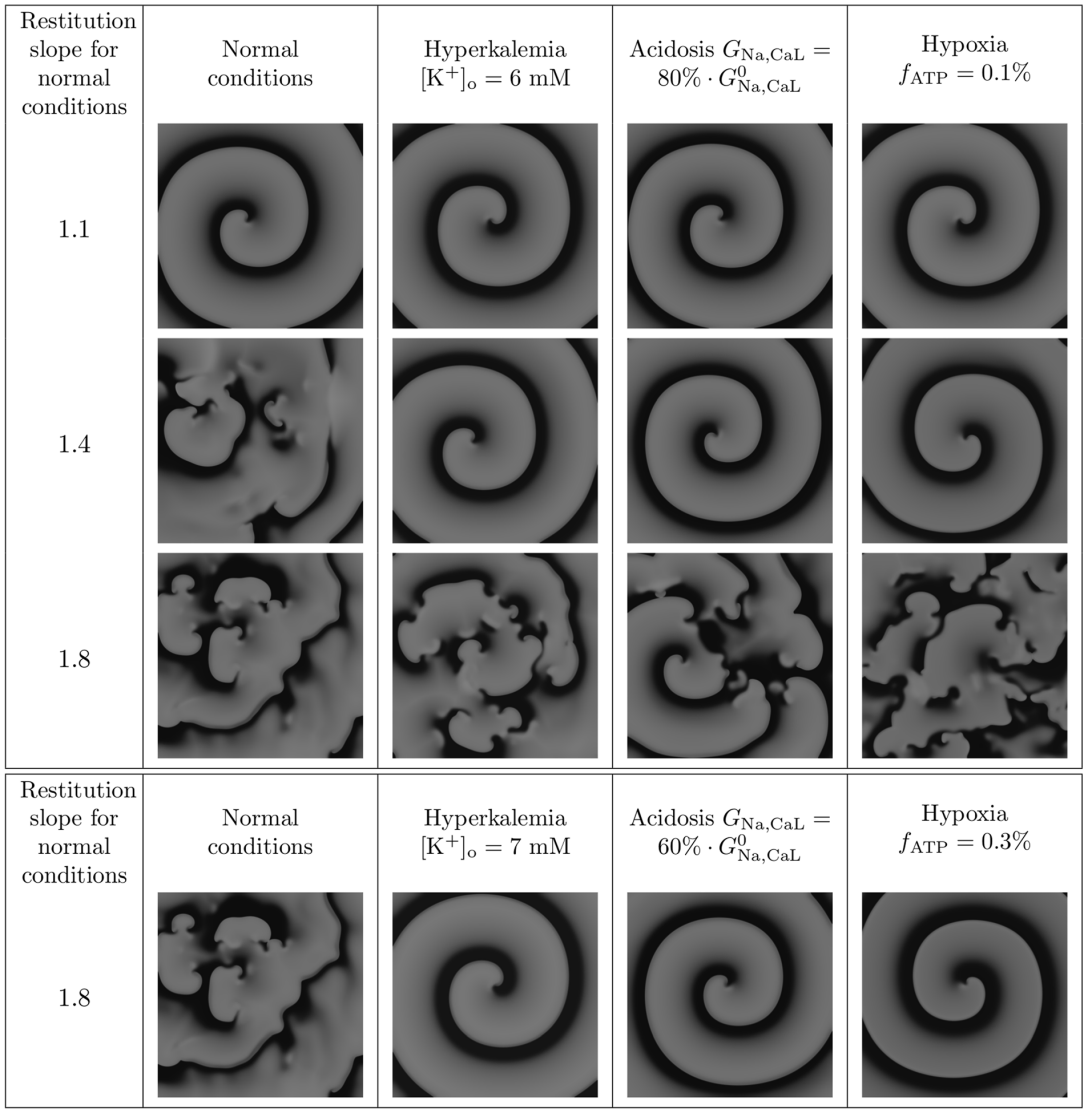
Stability of spirals under ischemic conditions. Greyscale shows membrane voltage, with light grey indicating depolarized tissue.

Figure 3 shows the resulting patterns of transmembrane voltage after 10 s of simulated activity for each parameter set and under each component of ischemia. For tissue with a restitution slope of 1.1 under normal conditions, the spiral remained stable regardless of ischemia. For a restitution slope of 1.4 (the second row), a complex fibrillatory pattern developed under normal conditions resulting from breakup of the initial spiral wave, however each ischemia factor prevented breakup. The third row gives an example for a relatively high restitution slope of 1.8 under normal conditions. In this case the spiral wave broke into fibrillation under both normal and ischemic conditions. However, it was still possible to stabilize the spiral rotation by further increasing the ischemia parameters. The bottom row in Figure 3 corresponds to that case: as ischemia became more severe the activation pattern did not evolve into fibrillation.

Overall, all three components of ischemia acted to stabilize re-entry, and to prevent the breakups that can happen under normal conditions. This is in line with the prediction that we derived from analyzing the restitution curves in the previous section, which was based on flattening of the restitution curves due to ischemia.

Our next step was to understand the change of the main dynamical characteristics of cardiac tissue with ischemia and to compare these with the activation patterns observed in the human heart [13]. Therefore, we performed simulations using an anatomically detailed model of the human ventricles developed in [21]. To explain our results we shall use information on 1D and 2D wave propagation collected in the previous sections.

### VF in an anatomical model of the human ventricles incorporating simulated global ischemia

#### Wave patterns

The three components of ischemia were modeled as described in Table 1. VF was induced using an S1S2 protocol which resulted in a subsequent spiral wave breakup and onset of the fibrillatory pattern as described in [21]. It was shown that this protocol reproduces the main features of VF in the human heart [21], [10]. The resulting state after 5 s of initialization was saved and used as an initial condition for subsequent simulations to investigate the effect of ischemic components on VF dynamics.

**Figure 4:**
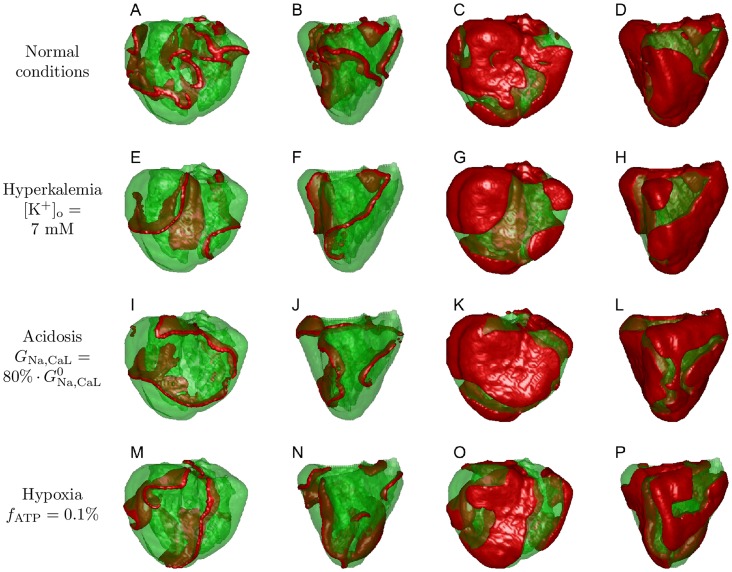
Fibrillation patterns. A–D: normal conditions, E–H: hyperkalemia, I–L: acidosis, M–P: hypoxia. In the first two columns the red color shows the location of wavefronts, and in the last two columns it shows exited tissue where the transmembrane voltage is higher than 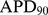 level. Columns 1, 3 show the frontal view and columns 2, 4 show the lateral view.

Figure 4 shows example snaphots of activation in the anatomically detailed model for both normal and ischemic conditions, with each ischemic component set to values corresponding to those obtained from the human recordings, as we will show later.

Figures 4A–D show typical excitation patterns during simulated VF, both in terms of wave fronts (A: front view, B: lateral view) and activated regions for which the voltage is higher than 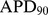 level (C, D). Here we have a complex fibrillatory pattern characterized by multiple re-entrant waves. For every subsequent simulation, we started from this initial state and gradually (within 200–800 ms) changed the corresponding parameters from their normal values to the values corresponding to the different ischemic conditions, and then computed for a further 15 s of model time. Examples of fibrillation patterns we obtained are shown in Figure 4E–P.

Figures 4E–H give the resulting picture for hyperkalemia with 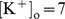 mM. The complexity of activation was slightly lower in comparison with the initial state, with fewer wavefronts. Figures 4G–H show the amount of activated tissue for this snapshot. The ratio of green to red was larger for hyperkalemia, which indicates that the tissue became less excitable. Similar results can be seen for hypoxia 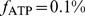 in Figures 4M–P. The medium was less excitable and the complexity of VF was slightly lower compared to normal.

Acidosis had a less pronounced effect on both excitability and pattern complexity. Figures 4I–L gives an example of fibrillation under acidosis conditions where the conductivity of 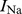 and 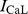 were reduced to 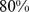.

#### Period of VF

The period of VF in each simulation was determined using a discrete Fourier transform of time series of transmembrane voltage obtained from 10000 locations in the heart model for the last 7 s of model time, and obtaining the period from the frequency of the largest peak in the spectrum.

We found that the period of fibrillation showed almost no dependence on acidosis. Even for a severe drop of 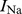 and 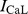 conductivity (5-fold) the change of the period was less than 5% compared to normal (not shown). This effect can be also seen on the restitution curves in Figure 2: the 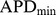 value, which is believed to determine the frequency of VF [10], is almost unchanged with acidosis. For this reason acidosis was excluded from further analysis and the following results describe a two parameter space of hyperkalemia and hypoxia.

**Figure 5:**
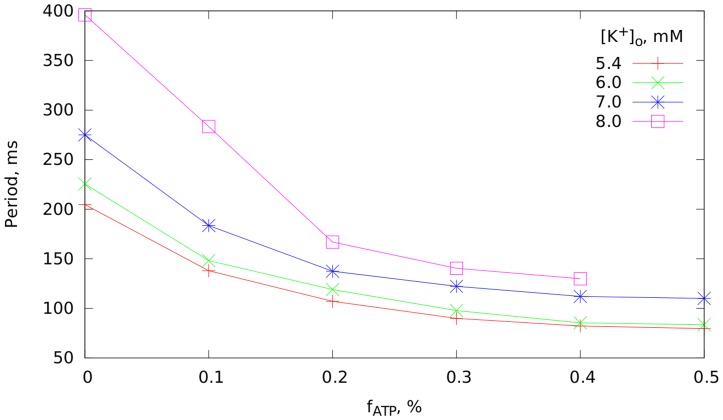
The effect of hyperkalemia and hypoxia on period of simulated VF in anatomically detailed 3D model.

The dependence of the average period of VF on hypoxia and hyperkalemia is shown in Figure 5. The period of fibrillation increased under hyperkalemia, and decreased under hypoxia. The effect of hyperkalemia was more pronounced for smaller values of 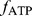. Also, a saturation effect can be seen: for larger values of 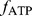 the effect of additional hypoxia was relatively small.

At the first glance, our observation that hyperkalemia acted to increase the period of VF contradicts the results of our 1D studies, which showed that APD decreased with increase of 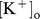. However, as in the case of acidosis, the most important factor here is 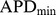, which increases with hyperkalemia (Figure 2A) and, therefore, explains the observed increase in the period of VF.

Explanation of the observed decrease of period under hypoxia is straightforward. The main effect of hypoxia is shortening of the action potential. Indeed, we see in Figure 2C that 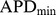 becomes smaller under hypoxia. This shortening of APD results in a decrease of the period of VF. The effect of hyperkalemia turned out to be more pronounced for smaller values of 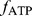.

We used the data of Figure 5 to estimate the effects of these two factors of ischemia on the period change during ischemia observed in [13].

### Estimation of the relative contribution of hyperkalaemia and hypoxia to patient data

**Figure 6:**
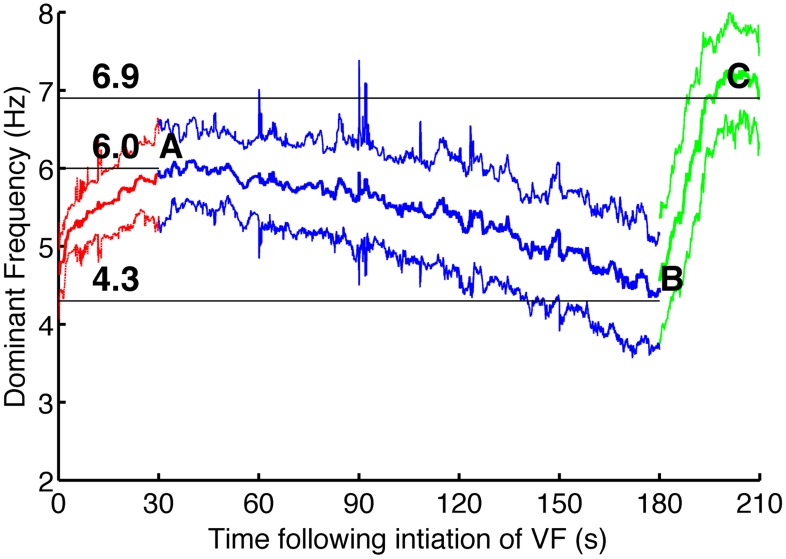
Change of DF over time for one patient (H066). Red denotes DF recorded with the heart perfused, blue denotes ischemia, and green reflow. The middle line shows mean DF, averaged over electrodes, and the top and the bottom lines show one standard deviation either side of the mean.

Our results allowed us to estimate the extent of hyperkalemia and hypoxia for each patient involved in the clinical study [13]. We used the dependency of how DF (1/period) changes throughout experimental VF for individual patients. One of these recordings is shown in Figure 6. The red region corresponds to normal perfusion of the heart, blue to global ischemia and the green to reperfusion. As one can see, DF gradually decreases during ischemia and then increases abruptly after reperfusion to an even higher level that it was initially.

For each patient we used three points on this graph: DF at the beginning of ischemia (30 s, point A), DF at the end of ischemia (180 s, point B) and DF at the end of the experiment (210 s, point C). Ischemia takes place in between points A and B, thus 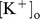 and 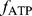 are increasing there. At point B we expect to have the highest values for these quantities.

At point B reperfusion starts, resulting in a rapid elevation of DF. Figure 5 indicates that hypoxia acts to shorten the VF period, thus it is likely to be responsible for this change. Therefore we assumed that the recovery of hypoxic 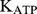 channels during reperfusion is slower than the recovery of extracellular potassium concentration. We assumed that 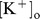 had returned to a normal value by the point C whereas 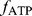 remains at the same level as at point B. Thus, knowing DF at point C, we can find the fraction of open 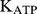 channels at that moment. Using this value and the value of DF we can estimate 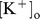 at point B. The results we obtained using this algorithm are given in first three columns of Table 2. The results shown in columns 4–7 of the table deal with a possible partial recovery of 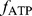 and will be described in the discussion section. The results in the last two columns are based on the assumption that 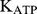 channels open even before the beginning of ischemia. This will also be described in the discussion section.

We see that the estimated 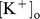 concentrations for different patients vary in the range from 6.5 to 8.0 mM, while the fraction of activated 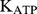 channels 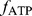 does not go beyond 0.1%.

**Figure 7:**
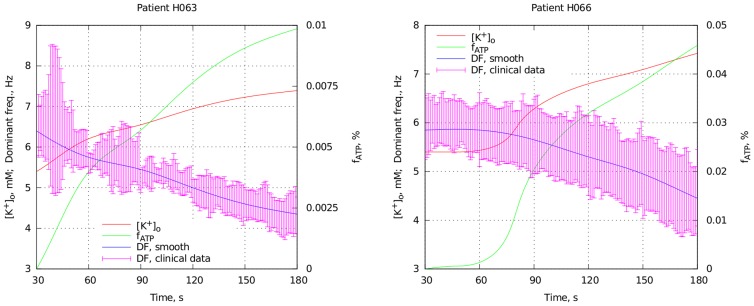
Two examples of how hypoxia and hyperkalemia evolve during course of ischemia. Pink: clinical data on the dominant frequency, averaged over 1 s window with standard deviation. Blue: smoothed curve used for the fitting. Red: extracellular potassium concentration. Green: fraction of activated 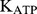 channels. The left vertical axis corresponds both to the extracellular potassium in mM and the dominant frequency in Hz. The right axis corresponds to the fraction of activated channels, measured in percents.

We also estimated how hypoxia and hyperkalemia change in the course of ischemia. This problem of fitting does not have a unique solution because the period of fibrillation depends on two parameters, while we have only one period dependency for each patient. To account for that we assumed that the dependencies of 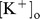 and 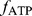 are monotonic with time, and these values can never decrease during ischemia. Then we wrote the relation between the rate of change of these values:
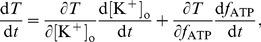
(5)where 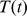 is the period of fibrillation over time (from the clinical results) and 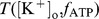 is the dependency of VF period on the ischemia components, obtained from Figure 5. Our goal was to determine the patient specific functions 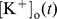 and 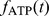, based on known 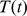, 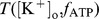 and boundary conditions from Table 2. To solve (5) we needed to impose an additional constraint on our functions. Thus we assume that
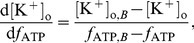
(6)where 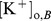 and 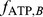 are the values we fitted for point B. This constraint ensured the values of hypoxia and hyperkalemia tended to reach those values at point B. The results we obtained using this approach for two patients are shown in Figure 7. The rest of the results for all 10 patients are available in Figures S1–S10. We see that we can fit the clinical data with smooth monotonic functions using our approach. However, as our constraint (6) cannot be justified from biological background, these fits can be considered as a conjecture rather than established results.

### Number of re-entrant sources

The study of [13] describes how the number of epicardial phase singularities and the number of wave fronts change during the course of ischemia. These values have approximately the same dynamics and to understand the results we studied the change in the number of phase singularities. Phase singularities are points where filaments intersect the surface of the heart [7], [10]. As was shown in [10], the number of filaments obtained from simulations in an anatomically detailed model of human ventricles provide a good estimate of the number of phase singularities on the surface of the heart. We counted the number of filaments in our model in normal conditions and under different factors of ischemia.

**Figure 8:**
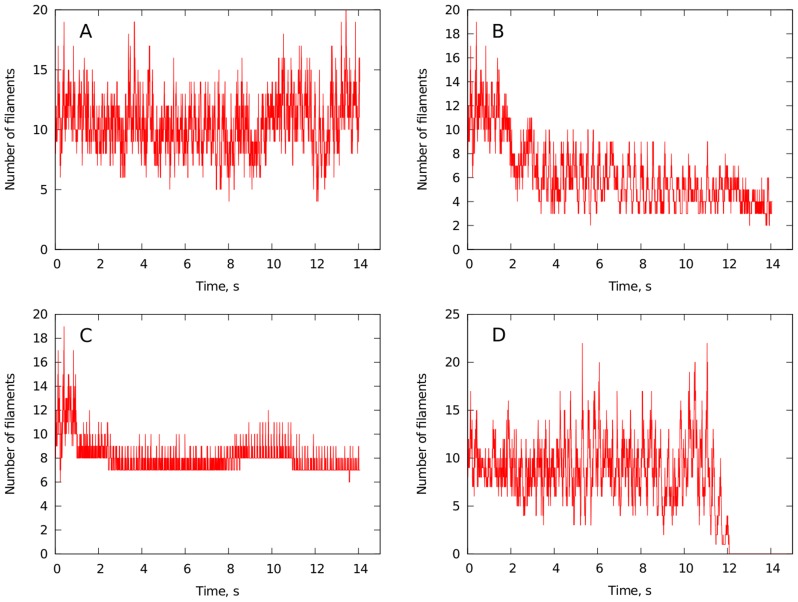
Change of the number of filaments in course of time under different ischemia factors obtained from numerical simulations. A: normal conditions, B: hyperkalemia, C: hypoxia, D: example of spontaneous fibrillation stop.

Figure 8 shows four examples of how the number of filaments depends on time under conditions that we expect to correspond to those observed for the first 2.5 minutes of ischemia. Figure 8A shows filament numbers during normal conditions. We see dynamics similar to that reported in [21]. Figures 8B and C correspond to hyperkalemia 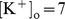 mM and hypoxia 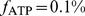, respectively. We see that compared to normal condition there is a tendency for decreasing of the number of filaments. Finally, Figure 8D gives an example where simulated VF terminated spontaneously, although the number of filaments stayed relatively high until immediately prior to termination. For that simulation we used 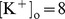 mM and 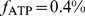.

**Figure 9:**
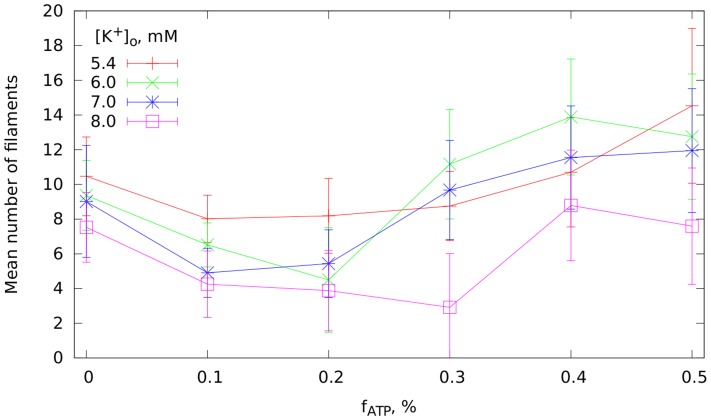
The mean number of filaments of a fibrillation pattern averaged over time of simulation after applying ischemic conditions. The error bars indicate the standard deviation of root mean square.

The mean number of filaments in the two parameter space—hyperkalemia and hypoxia—for a wider range of parameters is given in Figure 9. The error bars in this figure correspond to the standard deviations of root mean square. The point with 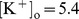 mM and 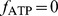 corresponds to normal conditions. We can see that for values of hypoxia 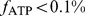 the complexity of the pattern slightly decreases. However, it increases for larger values of hypoxia. All these results differ substantially from our observation from the 2D patterns (see Figure 3). In 2D, we observed that every single component of ischemia suppressed breakup, which would correspond to a decrease of filament numbers and termination of fibrillation in our whole heart simulations. However, this figure shows that for 3D an increase in fibrillation complexity as the degree of ischemia increases.

This is a surprising result that can be explained in the following way. The 2D simulations always started from a single spiral, and breakup was self induced. In 3D, however, we started from an initial pattern with multiple re-entrant waves. This complex pattern was able to maintain itself in spite of the presence of simulated ischemia. To confirm this observation, we performed simulations in 2D, starting from a developed VF pattern. These simulations showed that even in 2D, the complex pattern can persist compared with initiation with the S1S2 protocol of Figure 3.

The results of the clinical research are presented in Table 3. The minimal and maximal value of the number of phase singularities on the surface of the heart are given there for both the beginning and the end of ischemia. As shown, that there are large deviations in PS numbers making it difficult for us to give conclusive statements from these data. However, they do indicate relative conservation on PS numbers during ischemia.

**Table 3:** Minimal and maximal values of the number of phase singularities during the first and the last ten seconds of ischemia for the patients studied in [13].

Patient	Before ischemia	After ischemia
H055	2–17	1–18
H057	2–15	2–20
H058	3–22	4–25
H059	2–22	3–26
H060	2–15	2–18
H062	4–22	5–24
H063	1–14	1–19
H064	1–13	2–15
H065	1–11	1–18
H066	2–18	2–22

If we now compare these results with results on filament numbers shown in Figure 9 we can conclude the following. In the range of ischemic components which we estimated for the individual patients (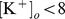 mM, 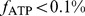) we see some tendency for a decrease in the filament numbers in our simulations. However, this decrease is minimal and to some extent can be considered as conservation of filament numbers in a way similar to that we see in clinical data. However, our simulations indicate that we can expect an increase in filament numbers if ischemia is more prolonged or if 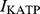 channels open to a greater extent.

## Discussion

The aim of this study was to use a model of cardiac electrical activity to gain insight into the mechanism underlying the organization of ventricular fibrillation observed in the in-situ human heart with global cardiac ischemia. We performed numerical simulations using a detailed electrophysiological model of cardiac cells and tissue under normal and ischemic conditions. This model was examined in pseudo-1D and 2D tissue geometries, and in an anatomical model of human ventricles which takes the geometry and anisotropy of cardiac tissue into account. As in [5], [20], [19] we subdivided the effects of ischemia into three main syndromes: hyperkalemia, acidosis and hypoxia. To represent the activation of 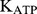 channels in hypoxia, we developed a new model of the human 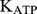 channel, based on recordings of [16].

Our 1D and 2D studies demonstrated that each component of ischemia results in a shortening of APD under the same base cycle length, which is in accordance with the results of other modeling research [19]. Our results for CV restitution (dispersion relation) are similar to those obtained in [20] with a small difference: the CV does not depend monotonically on hyperkalemia. As can be seen in Figure 2D, in our simulations CV increases with hyperkalemia for lesser values of 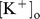 whereas for higher ones it decreases. This supernormal conductance effect was not observed in studies of [20], which may be due to a different stimulation protocol. Overall, the restitution curves we obtained are very similar to [14].

According to the restitution hypothesis, our observation that the steepness of APD restitution curves decreases with ischemia makes dynamical instabilities less likely to occur, and wavebreak leading to multiple wavelet VF less probable [26]. We confirmed this in our 2D simulations. In particular, we found that it was sufficient to increase any of the ischemic factors within their physiological range to prevent break up that can occur under non ischemic conditions. This is also in line with the simulations on effects of activation pattern due to restitution steepness by [26], [28].

The main part of the present study deals with simulations in a 3D anatomically accurate model of the ventricles of the human heart for situations that resemble the clinical set up used in [13]. Our aim was to describe how the organization of simulated VF is altered due to cardiac ischemia. We found that acidosis had almost no effect on the activation rate, while hyperkalemia decreased and hypoxia increased the frequency of VF. Studies of this effect were not previously performed in anatomical models, although they are similar to 2D simulations and experiments in animal models. The effect of hyperkalemia on the period of spiral wave rotation was studied by [29] who obtained similar dependency but only for concentraions of 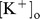 larger than 8 mM. The same effect of hypoxia was investigated by [30] on canine ventricular slices and later by [31] on swine hearts. The results obtained in their experiments are in accordance with our simulations.

The dependence of activation rate on ischemic factors could be predicted by looking at our APD restitution curves. Fibrillatory excitation patterns have a very high activation rate, and thus the leftmost regions of restitution curves are of considerable importance since they correspond to high activation rate and short diastolic interval. If we look, for instance, at Figure 2A we see that the leftmost points shift to the right with hyperkalemia. Thus the activation frequency of fibrillation decreases. The minimal possible values of APD, which are given by the left most points, are called 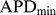. Thus this finding once again emphasizes a particular importance of these 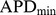 on VF organization which has also been demonstrated in other studies [23], [27]. Note, that the 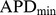 point is achieved at the minimal possible period of stimulation, which is, perhaps, more directly related to the period of excitation during VF.

With our 3D simulations we were able to construct a model of the dependency of VF period on both hyperkalemia and hypoxia. This model, along with additional assumptions about the relative rates at which the different components of ischemia develop allowed us to propose a way to estimate the extacellular potassium concentrations and the fraction of open 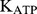 channels for the recordings in the clinical study [13]. The estimated external potassium concentrations correspond to experimental data showing a rate of 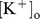 change of about 0.5–1 mM per minute [2].

The most important assumption in this study was that the rate of recovery from hypoxia during reperfusion is slower than the rate of recovery from hyperkalemia. Experimental data from porcine hearts [32] show that the complete recovery of 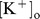 during reflow can occur within less than one minute. Unfortunately the data available on the recovery of 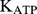 are controversial which makes it difficult to assess whether our assumption is correct. Data from the the human heart [13], [33] obtained at the whole organ level show that, even after 5 minutes of reflow, less than 70% of the APD shortening (which was assumed to be an effect of 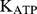 opening) had been reversed. On the other hand, experimental data from single cells demonstrate that recovery can be very fast, of the order of a few seconds [34], [35].

To investigate an effect of this possible partial recovery of hypoxia we estimated how our estimates change if we assume that after 30 seconds of reperfusion either 25% or 50% of 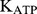 had been recovered. These estimates are shown in the columns 4–7 of Table 2. The second and the third columns are given for the case of no recovery. As we can see, partial recovery does not have a pronounced effect on the estimated values of hyperkalemia during ischemia. Indeed, for most of the patients the error in 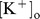 estimation due to this partial recovery is about 0.3–0.4 mM.

We have also proposed a method of fitting the change in 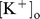 and 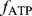 for each individual patient during global cardiac ischemia. Note, however, this problem is nontrivial because its exact solution requires more experimental details on the speed of various processes during ischemia. The approach that we followed is based on solving a boundary problem for an ordinary differential equation (ODE), expanded with one artificial ODE that provided a relation between relative change of the hyperkalemia and hypoxia factors. We also tried other, less sophisticated methods to target this problem, such as linearly varying one of the factors and finding the time course for the other one. The deficiency of these simple methods was that they violated the assumption that ischemia factors grow monotonically for some of the data. Therefore we followed our approach, although it is not based on any mechanistic background. As soon as more data on the effects of ischemia in the human heart are available, our approach could be improved by adding more constraints on the solution.

**Figure 10:**
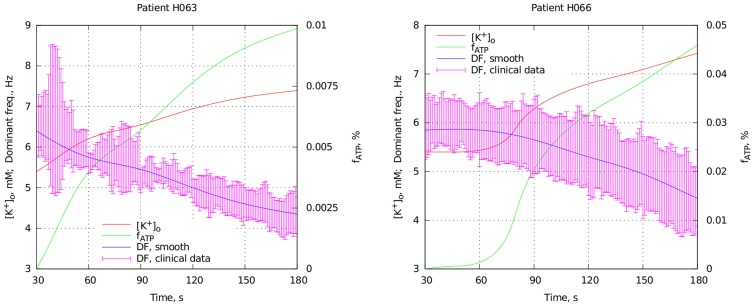
Two examples of how hypoxia and hyperkalemia evolve during ischemia assuming that the 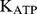 channels open before the beginning of ischemia due to energy depletion. The legend is the same as in Figure 7.

Our clinical data show that in all recordings there was an initial increase of the dominant frequency that happens during the first 30 s of the study under normal perfusion. This effect was also obtained in other studies [9], [36]. A possible explanation for this effect is the following. Since ventricular fibrillation is an energy consuming process, it may shift the 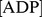 to 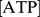 ratio, and hence diminish the energy capabilities of the cells. This would lead to opening of 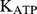 channels, and result in an increased DF. To examine this possible explanation, we modified our fitting method, taking the DF at the beginning of the recording as corresponding to normal conditions. The maximum values for hypoxia and hyperkalemia for this case are shown in the last the columns 8–9 of Table 2. We see that this alternative assumption leads to an increased number of activated of 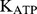 channels, whereas the extracellular potassium concentration remains relatively unchanged. We also adapted this assumption for our fitting algorithm describing how hypoxia and hyperkalemia evolve in the course of ischemia. The results for two patients are given in Figure 10.

Finally, we studied the effects of global cardiac ischemia on the complexity of activation patterns during simulated VF. For that we used an established VF activation pattern as an initial condition for the simulations. This type of initial condition differs from most of the previous studies of the restitution based fibrillation, where a single spiral is used for the initial condition and the conditions under which this spiral develops into fibrillation are in question. We found that using established VF as an initial conditon showed that flattening of the restitution curve caused by ischemia does not usually lead to VF termination, but rather results in a small number of stable re-entrant sources. We think that this finding does not contradict to the results obtained on spiral breakups in 2D. Indeed, flattening of restitution curves just prevents the onset of VF, however, this process is not directly related to the elimination of multiple existing spirals. In fact, flattening of the restitution curves decreases dynamical instabilities and makes the rotation of spirals more stable, thus it is even more likely to stabilize multiple spirals in the tissue. The tendency for a fibrillation pattern to become more organized due to global ischemia was first demonstrated experimentally by [37] on rabbit hearts. In addition, a recent experimental study on the effects of hypoxia in swine hearts by [31] has also shown that activation of 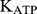 channels makes the fibrillation pattern more stable and stops rotors from meandering. However, a study by [26], performed also on swine hearts, argues that flattening of the restitution curve does favour VF termination, though, to achieve that flattening they used a potassium channel antagonist—bretylium—which has an effect opposite to hypoxia.

### Limitations

There are several factors that were not taken into account in this study. We used a homogeneous model of the heart which does not take into account the differences between epicardial, endocardial and M-cells. However, as it is shown in [38] the differences between these cell types decrease substantially at the high frequency of excitation typical for VF patterns. Note that the differences between different cell types are not that pronounced in tissue due to the electrotonic effect. Next, we used a monodomain model, to represent the cardiac tissue. Bidomain models are of considerable importance when describing defibrillation phenomena but in the absence of applied external currents the difference between these two types is extremely small [39].

Due to numerical limitations, we were not able to reproduce full 3.5 minutes of the clinical study in our model and limited ourselves by only 20 seconds. We did not check if the effects we studied are also presented in other detailed electrophysiological models of cardiomyocyte, such as [40] and [41], nor did we take into account effects due to mechanical activity of the heart during VF.

We did not consider the change in the gap junction conductance caused by ischemia. There are several experimental studies on animal models that demonstrate that the effect on gap junction uncoupling is relatively small in first minutes of ischemia. The investigations in a rabbit papillary muscle [42] show that under ischemic conditions the intracellular resistance stays constant within the first 10–15 minutes, whereas the extracellular resistance slightly changes immediately following the onset of ischemia. However this change was not associated with a significant decrease of the conduction velocity within the first 4 minutes of ischemia. Another result obtained in isolated rat hearts [43] indicates that the absolute value of the impedance of the tissue changes for about 3 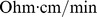 with the initial value of about 150 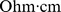. Finally, another set of experiments on rabbit papillary muscle [44] also shows that the tissue resistance stays remarkably stable for the first 10 minutes of acute ischemia. These data allow us to assume that the process of gap junction uncoupling does not have a significant effect on the conduction velocity for the time scale of 2.5 minutes of ischemia.

We did not consider either the heterogeneity of the tissue response to ischemia nor heterogeneity in ionic channels distributions. As shown in other studies [45], heterogeneities in 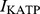 distribution can lead to a transmural gradient of the DF in myocardium. There are several studies that show that this gradient occurs in experimental models [11], [12], [46]. Furthermore, global ischemia may lead to different depths of ischemia in different regions of the heart, since the endocardium is in contact with a large volume of oxygenated blood in the ventricular cavities, at least in the early stages of VF. We expect that these heterogeneities can lead to a different fibrillation mechanism (to so-called mother rotor fibrillation) as well as increase in the number of filaments [47], [48]. This factor may also contribute to increasing the number of phase singularities localized at the surface of the heart.

The role of Purkinje system was not considered in this study. There are several experimental studies in dog hearts [46], [49], which show that the Purkinje system can play an important role in early termination of VF because it acts to increase the transmural activation rate gradient. However, a computer modeling study [45] shows that this effect is not that prominent in comparison with the effect of heterogeneity in 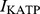 distribution.

Finally, we omitted several other effects that may occur during ischemia. In our case, depletion of ATP results only in activation of the 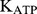 channel. We did not consider the effect of ATP on other important processes, such as functioning of the 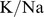 pump, the processes occurring at the mitochondria membranes and their effect on calcium dynamics. We did not take into account any changes of cell size or extracellular medium size occurring as a result of osmosis, and possible changes of the ionic concentrations due to it. It would be valuable to extend existing cell models to incorporate a more detailed description of ischemia, and to study the effects of ATP depletion using such a model.

## Supporting Information

Figure S1Evolution of hypoxia and hyperkalemia in the course of global ischemia for the patient H055 in [13], estimated by our fitting method. Pink: clinical data on the dominant frequency, averaged over 1 s window with standard deviation. Blue: smoothed curve that we used for our fitting. Red: extracellular potassium concentration. Green: fraction of activated 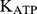 channels.(TIF)

Figure S2Evolution of hypoxia and hyperkalemia in the course of global ischemia for the patient H057 in [13], estimated by our fitting method. Pink: clinical data on the dominant frequency, averaged over 1 s window with standard deviation. Blue: smoothed curve that we used for our fitting. Red: extracellular potassium concentration. Green: fraction of activated 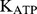 channels.(TIF)

Figure S3Evolution of hypoxia and hyperkalemia in the course of global ischemia for the patient H058 in [13], estimated by our fitting method. Pink: clinical data on the dominant frequency, averaged over 1 s window with standard deviation. Blue: smoothed curve that we used for our fitting. Red: extracellular potassium concentration. Green: fraction of activated 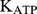 channels.(TIF)

Figure S4Evolution of hypoxia and hyperkalemia in the course of global ischemia for the patient H059 in [13], estimated by our fitting method. Pink: clinical data on the dominant frequency, averaged over 1 s window with standard deviation. Blue: smoothed curve that we used for our fitting. Red: extracellular potassium concentration. Green: fraction of activated 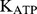 channels.(TIF)

Figure S5Evolution of hypoxia and hyperkalemia in the course of global ischemia for the patient H060 in [13], estimated by our fitting method. Pink: clinical data on the dominant frequency, averaged over 1 s window with standard deviation. Blue: smoothed curve that we used for our fitting. Red: extracellular potassium concentration. Green: fraction of activated 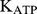 channels.(TIF)

Figure S6Evolution of hypoxia and hyperkalemia in the course of global ischemia for the patient H062 in [13], estimated by our fitting method. Pink: clinical data on the dominant frequency, averaged over 1 s window with standard deviation. Blue: smoothed curve that we used for our fitting. Red: extracellular potassium concentration. Green: fraction of activated 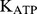 channels.(TIF)

Figure S7Evolution of hypoxia and hyperkalemia in the course of global ischemia for the patient H063 in [13], estimated by our fitting method. Pink: clinical data on the dominant frequency, averaged over 1 s window with standard deviation. Blue: smoothed curve that we used for our fitting. Red: extracellular potassium concentration. Green: fraction of activated 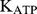 channels.(TIF)

Figure S8Evolution of hypoxia and hyperkalemia in the course of global ischemia for the patient H064 in [13], estimated by our fitting method. Pink: clinical data on the dominant frequency, averaged over 1 s window with standard deviation. Blue: smoothed curve that we used for our fitting. Red: extracellular potassium concentration. Green: fraction of activated 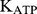 channels.(TIF)

Figure S9Evolution of hypoxia and hyperkalemia in the course of global ischemia for the patient H065 in [13], estimated by our fitting method. Pink: clinical data on the dominant frequency, averaged over 1 s window with standard deviation. Blue: smoothed curve that we used for our fitting. Red: extracellular potassium concentration. Green: fraction of activated 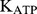 channels.(TIF)

Figure S10Evolution of hypoxia and hyperkalemia in the course of global ischemia for the patient H066 in [13], estimated by our fitting method. Pink: clinical data on the dominant frequency, averaged over 1 s window with standard deviation. Blue: smoothed curve that we used for our fitting. Red: extracellular potassium concentration. Green: fraction of activated 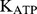 channels.(TIF)
